# Response of a three-species cyclic ecosystem to a short-lived elevation of death rate

**DOI:** 10.1038/s41598-023-48104-6

**Published:** 2023-11-25

**Authors:** Sourin Chatterjee, Rina De, Chittaranjan Hens, Syamal K. Dana, Tomasz Kapitaniak, Sirshendu Bhattacharyya

**Affiliations:** 1 Department of Mathematics and Statistics, Indian Institute of Science Education and Research, Kolkata, West Bengal 741246 India; 2 Department of Physics, Raja Rammohun Roy Mahavidyalaya, Radhanagar, Hooghly 712406 India; 3 Center for Computational Natural Sciences and Bioinformatics, International Institute of Information Technology, Gachibowli, Hyderabad 500 032 India; 4 Division of Dynamics, Faculty of Mechanical Engineering, Lodz University of Technology, 90-924 Lodz, Poland; 5 Centre for Mathematical Biology and Ecology, Department of Mathematics, Jadavpur University, Kolkata, 700032 India

**Keywords:** Ecological modelling, Nonlinear phenomena

## Abstract

A balanced ecosystem with coexisting constituent species is often perturbed by different natural events that persist only for a finite duration of time. What becomes important is whether, in the aftermath, the ecosystem recovers its balance or not. Here we study the fate of an ecosystem by monitoring the dynamics of a particular species that encounters a sudden increase in death rate. For exploration of the fate of the species, we use Monte-Carlo simulation on a three-species cyclic rock-paper-scissor model. The density of the affected (by perturbation) species is found to drop exponentially immediately after the pulse is applied. In spite of showing this exponential decay as a short-time behavior, there exists a region in parameter space where this species surprisingly remains as a single survivor, wiping out the other two which had not been directly affected by the perturbation. Numerical simulations using stochastic differential equations of the species give consistency to our results.

## Introduction

Coexistence of diverse biological organisms is essential to maintain a stable ecosystem. An extinction of any species can jeopardize biodiversity and hence cause a threat to the survival of other natural inhabitants. Hence, understanding various intraspecies and interspecies interactions are the most significant part of studying evolutionary dynamics. It has been the most challenging part of the study because of its diversity and complexity. Again the stability thus obtained is not robust at all and may be disrupted at times due to a small change in any of the controlling parameters. The study of transients as well as long-time dynamics following such disturbances in an ecosystem is itself an important area of study because nature frequently faces numerous perturbations that are potential threats to the stability of the ecosystem.

From various reports^1,2^ it has been assumed that the competitive interactions among various species in a community are the determining factor for a community structure. However, many ecologists have argued that interspecific competition among existing species does not play a major role in ecological diversity, but disturbances such as storms, floods, drought, pandemics, etc. are important events that control community diversity^3–5^. So it becomes important to understand the system’s response to disturbances to understand the fate of biodiversity. The relationship between the disturbances and their consequence on species diversity has been addressed by ecologists empirically^6–8^ and also from a theoretical point of view^9–13^ over a long time. According to the intermediate disturbance hypothesis (IDH)^14,15^ diversity is high for intermediate disturbance and the disturbance-diversity relationship (DDR) shows a peak response. But related empirical studies^7^ show positive, negative, and U-shaped DDR in nature. Miller et al.^16^ in their model based on stochastic finite difference equations showed that increasing, decreasing, or U-shaped DDR can be recognized, and each of the shapes will depend only on different aspects (such as intensity, duration of disturbance, and timing) of the disturbances being used. Hastings^17^ showed for predator-prey systems that the long transient dynamical analysis is more useful for explaining the persistence of the system. Several studies^18–21^ have been done on the response of ecosystems in the presence of disturbances by considering the various dimensions of stability of the system. Kondoh^10^ through his simple model explained how the interaction of productivity and disturbances control the diverse pattern of species abundances, which was obtained from empirical studies. Characterizing the disturbances as pulse and press Inamine et al.^22^ have analyzed the effect of pulse and press disturbances on asymptotic and transient community dynamics of the Lotka-Volterra model for understanding their implications on species coexistence. Holt^23^ has shown for a competitive Lotka-Volterra system in the presence of disturbances, the competition being reduced may lead to the extinction of some of the competing species. Although the disturbances are most often viewed as a key factor for reducing ecological divergence there are reports^24^ that suggest the coexistence of many similar species in the presence of environmental uncertainty. In another report^25^, authors have shown the effect of intra- and interspecies epidemic spreading on species circumstances where species are considered mobile. The results are substantiated by theoretical interpretation based on a nonlinear differential equation.

The motivation of our present work is to study the transient and long-time dynamics of a simple three-species model ecosystem perturbed by a disturbance. The long-time dynamics (i.e. dynamics at a large time after the perturbation is gone) is particularly important because it reflects the fate of the ecosystem: Whether it would be able to preserve the coexistence or not. Earlier, the issue of maintaining biodiversity has been addressed mathematically by different models based on the game theory and statistical mechanics^26–29^. Among various models, the cyclically interacting rock-paper-scissor (RPS) model has been widely studied^30–40^ and is found to provide more insights on the mechanism of coexistence of species^41–44^. Various reports have been published on this RPS model focusing on spatial pattern formation^45–48^, the impact of mobility^49–55^, the effect of mutation^56–58^ to check coexistence. Using this RPS model in the May-Leonard formalism we tried to explore the dynamics of coexisting species after applying a pulse of increment in death rate on one of the species in the presence of other non-variable interactions. The idea behind considering an increase in death rate for a finite period lies in the fact that an ecosystem is often affected by catastrophic events like epidemics or any natural disaster that spike up the deaths of some selected species. For example, Plague hit the mammals only^59^, avian influenza affects only birds^60^, and COVID-19 has increased the death rate of humans for a finite amount of time^61^. The death rates of the corresponding species generally come down to normal (lower) values once the effects of the diseases are over. Even in the case of environmental impacts, species are seen to be affected selectively sometimes. Lately there have been some significant works on mimicking the impact of the environment on the population dynamics^62–64^. One of these works has incorporated the environmental effect by random switching of the reproduction-predation rate of a particular species between two values^62^. Periodic and random switching of another form of environmental impact have also been considered^63^. In our present work, we have considered only one pulse-like change in the death rate of one particular species instead of random or periodic switches. Again in the context of real-world phenomena, there are reports on some sub-species of the coral reef in the ocean’s ecosystem that are found to be selectively vulnerable to the effect of marine heatwaves^65,66^. These examples indicating that one or a few species can face a sudden increase in death rates for a finite time in an ecosystem makes our model relevant, particularly in the context of epidemic outbreaks and mortality occurring in endangered species. The theoretical model thus could help in understanding and predicting the community outcome such as how much the species itself or the entire ecosystem is affected by the disturbance. We have calculated the probability of existence of each species under varying conditions (e.g. with varying strength and duration of the pulse or disturbance). Against an intuitive guess or expectation that coexistence goes at stake with increasing pulse strength and duration, we find a narrow region of the parameters where the particular species vulnerable to the disturbance surprisingly survives alone while pushing the other two to extinction. This phenomenon is counter-intuitive to our common perception of the response of an ecosystem under a threat. In addition, we find an exponential decay of the population density of the endangered species within the persistence period of the disturbing pulse on the death rate.

In Sec. 2 we describe the model, the protocol, and the method of Monte-Carlo simulation executed on the system. We present the outcomes and also an analysis of the same through the numerical solution of stochastic differential equations in Sec. 3. Finally, conclusive remarks on the results are made with an outlook in Sec. 5.

## Model and simulation

The stochastic dynamics of the system are studied primarily by Monte Carlo simulation mapping the entire system to a 2-dimensional square lattice with periodic boundary conditions applied. The sites of the square lattice are either occupied by any of the three different species - $A,\;B,\;C$ or remain vacant. The constituent species have corresponding predation rates, $p_a,\;p_b,\;p_c$ operating in a cyclic manner. In addition, the elements of the species have reproduction rates, $r_a,\;r_b,\;r_c$, and death rates, $d_a,\;d_b,\;d_c$. Here the term death may be interpreted as natural or accidental death for which no other member of the system is responsible. We adopt the May-Leonard formulation^32^ where the total number of individuals is not conserved. Following this formulation we have also assumed that, in the process of evolutionary game dynamics, the predation strategy can create a vacant site in the adjacent neighbor whereas the reproduction replaces a vacant site with an individual^67^. Therefore, if the normalized species abundance of *A*, *B* and *C* are $\rho _a$, $\rho _b$ and $\rho _c$ respectively, the conservation rule will be $\rho _a + \rho _b + \rho _c + \rho _v = 1$, with $\rho _v$ being the fraction of vacant site with respect to the total number of sites. In the cyclic process, we can write the predation strategy with the following set of interactions

1 $$\begin{aligned} \lbrace A, B, C \rbrace + \lbrace B, C, A \rbrace \longrightarrow \lbrace A, B, C \rbrace + V \text{ with } \text{ rate } p_{\lbrace a, b, c \rbrace } \end{aligned}$$

where V denotes a vacant site. Apart from the predation factor, each species may generate its own offspring. The reproduction equations may therefore be written as

2 $$\begin{aligned} \lbrace A, B, C \rbrace + V \longrightarrow 2\;\lbrace A, B, C \rbrace \text{ with } \text{ rate } r_{\lbrace a, b, c \rbrace } \end{aligned}$$

In addition, our assumption of natural death may be represented by the following equations.

3 $$\begin{aligned} \lbrace A, B, C \rbrace \longrightarrow V \text{ with } \text{ rate } d_{\lbrace a, b, c \rbrace } \end{aligned}$$

Now we consider a situation when in a state of coexistence, one species (*A*) suddenly faces an increase in death rate for a finite period of time. It can be written as

4 $$\begin{aligned} d_a (t) = \left\{ \begin{array}{ll} d_a + \Delta d \;\; &{}\text{ for } 0\le t\le \tau \\ d_a \;\; &{}\text{ for } t> \tau \end{array} \right. \end{aligned}$$

**Figure 1:**
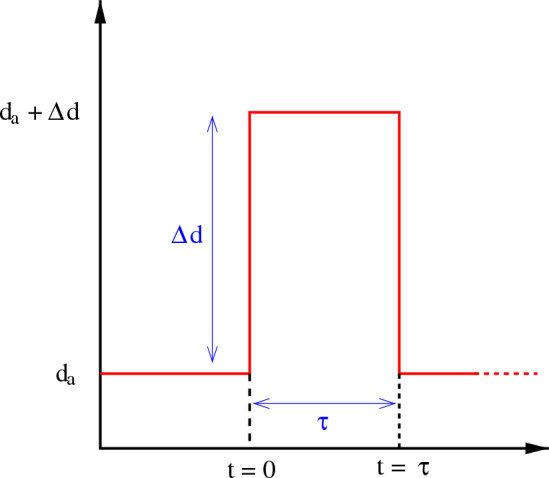
Schematic diagram of the death-pulse applied on species *A*. The elevated death rate remains active for the time duration $\tau$.

The situation may be imagined as the species *A* encountering a pulse of death rate for a duration of time $\tau$ (Fig. 1). We perform Monte-Carlo simulation of the system under this protocol. The simulation starts from a randomly chosen initial densities of three species ($\rho ^0_a$, $\rho ^0_b$, $\rho ^0_c$) with the constraint $\rho ^0_a + \rho ^0_b + \rho ^0_c + \rho ^0_v = 1$ where $\rho ^0_v$ is the initial density of vacant sites. At each Monte-Carlo step, an individual sitting on a site can interact with any of the four nearest neighbors in the von Neumann neighborhood. The simulation starts with a random selection of a non-empty site and one of its four nearest neighbors. The two sites perform predation-prey interaction with probability, $p_{a,b,c}$ for a non-empty nearest neighbor. If the nearest neighbor is found to be empty, the fellow in the primary site attempts reproduction with probability, $r_{a,b,c}$. In addition to these two possible actions, according to Eq. (3), the individual residing in the primary site may also die with a probability, $d_{a,b,c}$ making the corresponding site vacant. At each Monte-Carlo step the algorithm checks the probability of death first and then, if failed, it moves to predation or reproduction. However the final result would not change if this order of actions is altered. The entire process i.e. the Monte-Carlo step is repeated for a large number of times until the desired equilibration is reached. The time unit of our calculation is defined by *N* Monte Carlo steps, where $N\;(= L\times L)$ is the total system size. For the entire work, we have assumed $p_{a,b,c}=p$, $r_{a,b,c}=r$ and $d_{a,b,c}=d$.

## Results

As mentioned in the previous section, all the three species are supposed to start their journey from a coexisting state and therefore we choose a convenient set of parameters for which the system shows coexistence of all the constituent species^68,69^. Here, for a coexisting state, the densities of all the species oscillate around an average value. In our present work, we have taken $p = 0.2$, $r = 0.4$, $d = 0.1$ and the resulting densities ($\rho _a$, $\rho _b$, $\rho _c$) oscillate around 0.21. At the moment a death pulse of strength $\Delta d$ is administered on *A*. We set $t=0$ here. The pulse then stays for time $\tau$ which is referred sometimes to as the pulse width. The natural death rate of species *A* returns to $d_a\; (= d)$ after the death pulse is over (see Fig. 1). Given this situation, we study the long-time dynamics of the system to find the effect of the pulse. The system size taken here is $N=200\times 200$.

**Figure 2:**
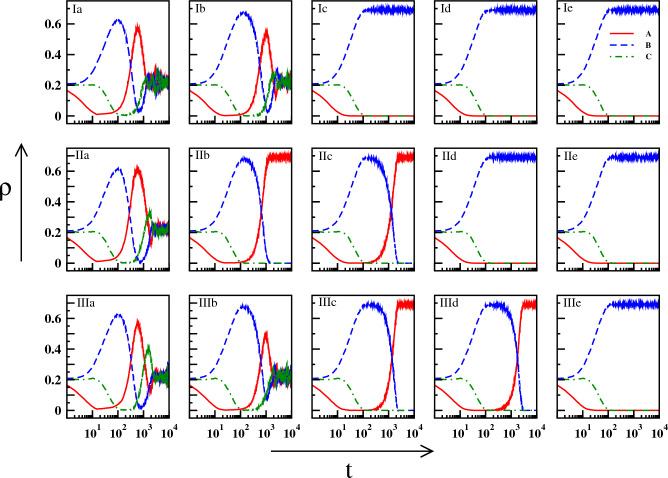
Real time dynamics of the densities of *A* (red line), *B* (blue dashed line), and *C* (green dot-dashed line) for different initial configurations and different $\tau$, all having $\Delta d = 0.2$: Three rows (I, II, III) are for three different initial configurations, and five columns (a - e) are for five different values of $\tau$: $15,\; 25,\; 35,\; 45,\; 55$.

The detailed long-time dynamics after the application of the pulse is reflected in Fig. 2. The plots are for three different initial configurations (I, II, III). For each configuration, the densities of the three species have been investigated with increasing pulse-width, keeping the strength of the pulse fixed at $\Delta d = 0.2$. In Fig. 2, Ia and Ib show coexistence of the species with small amplitude oscillation in the long run. As the duration of the pulse increases, Ic, Id, and Ie show the existence of the single species *B* only. A different initial configuration generates coexistence for $\tau =15$ in plot IIa of Fig. 2. For the same configuration, further increment of $\tau$ results in the single species existence of *A* first (IIb and IIc) and then, of *B* (IId and IIe). Another initial configuration shows coexistence for $\tau =15,\; 25$ (IIIa and IIIb respectively), the existence of single species *A* for $\tau =35,\;45$ (IIIc and IIId) and that of *B* for $\tau =55$ onwards (IIIe). This kind of appearance i.e. coexistence or existence of only *A* or *B*, has been found over time for different initial configurations as well. Nevertheless as we go on increasing $\tau$ in different configurations, the final scenario is always the same where the single species *B* exists. The result also remains the same for different sets of {*d*, *r*, *p*} which produce coexistence. We have demonstrated another result for a different value of *r* in Figure S1 of the Supplementary Material.

**Figure 3:**
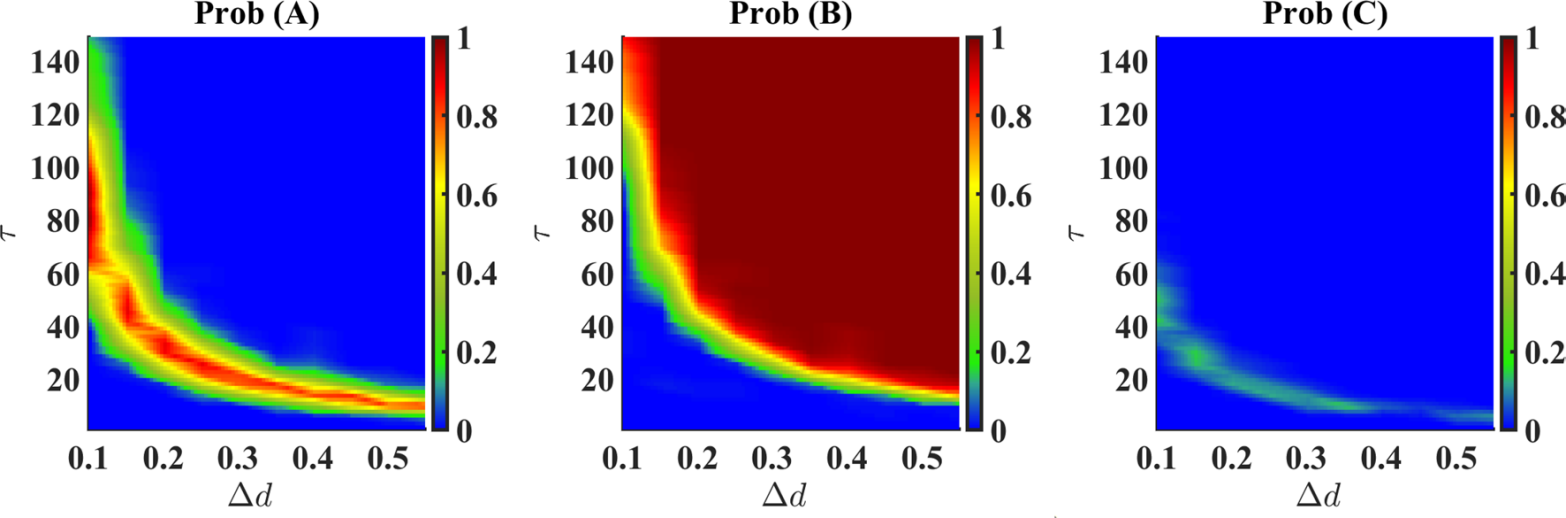
Probabilities of occurring (*A*, 0, 0) or (0, *B*, 0) or (0, 0, *C*) as a final state on $\Delta d - \tau$ plane. Large value of pulse and pulse duration steers the system such a way that only B survives (see red color in middle panel). Note that, for all cases, if the pulse or pulse duration is significantly high, the C species can never outperform the species *A* or *B* (right panel).

We have explored the effective region on the $\Delta d - \tau$ plane to investigate the probabilities of existence of all the species as presented in Fig. 3. The figures explain the probability of survival of a species with variations of $\Delta d$ and $\tau$. For high $\Delta d$, the survival of species *B* is observed to occur at small $\tau$ values. Hence it is possible to figure out different transition probabilities of any species with $\tau$ corresponding to a particular pulse height. The dominance of species B is also confirmed by this figure.

The appearance of the state (*A*, 0, 0), as observed in Figs. 2 and 3, even after the death pulse is applied to the species *A* is intriguing. This apparently counter-intuitive result occurs as an aftermath of certain values of $\Delta d$ and $\tau$, starting from different initial configurations generated randomly. However, the reason behind this occurrence is that for those particular combinations of parameters, species *C* gets abolished very fast, leaving behind a very small amount of species *A* and a considerably large amount of species *B*. As the death rate of *A* decreases (from $d+\Delta d$ to *d*) and *A* also predates *B*, the density of *A* grows eventually and that of *B* diminishes. As a result, *B* is abolished at a point in time and *A* becomes the lone survivor. In other cases of single-species survival where species *B* only exists, the dynamics after the abolition of *A* reveal quicker decay of species *C* than that of species *B* at higher $\tau$ values.

### Analysis with stochastic differential equation

In addition to the Monte Carlo simulation, we verify our results using stochastic differential equations using Euler’s method with the reproduction parameter having an intrinsic noise to add the variability in the system. We use Euler’s method because of the action of generating a random number from the distribution at each time point, making the function non-differentiable. Hence, we have assumed the discrete-time system, and on each step, all the random numbers are drawn independently. The simulation started with initial condition (0.3, 0.3, 0.3) and all other identical rates and after some time we applied additional death pulse with a depth $\Delta d$ for a time period of $\tau$ and after that, we set $\Delta d =0$.

In the Monte-Carlo simulation, the time evolution of the system before the commencement of the pulse was absent. Instead, the initial densities were taken to be ($\rho ^0_a$, $\rho ^0_b$, $\rho ^0_c$) which were the densities the system would have arrived for $r_a=r_b=r_c=r$, $p_a=p_b=p_c=p$ and $d_a=d_b=d_c=d$. The scenario is as if an ecosystem reaches a coexistent state ($\rho ^0_a$, $\rho ^0_b$, $\rho ^0_c$) for a particular set of reproduction, predation and death rates, and then species *A* suddenly faces an increase in the death rate for a time duration $\tau$. Thus both schemes present equivalent pictures. Following the deterministic equations^30–32^, the stochastic differential form of the rate equations mimicking this perturbation can be written as

5 $$\begin{aligned} \dfrac{d\rho _a}{dt}= & {} \rho _a(t) \left[ (r+0.05\mathscr {N}(0,1)) \rho _v(t)- p\rho _c(t)-(d+d')\right] \nonumber \\ \dfrac{d\rho _b}{dt}= & {} \rho _b(t) \left[ (r+0.05\mathscr {N}(0,1)) \rho _v(t)- p \rho _a(t)-d\right] \nonumber \\ \dfrac{d \rho _c}{dt}= & {} \rho _c(t) \left[ (r+0.05\mathscr {N}(0,1)) \rho _v(t)- p \rho _b(t)-d\right] \end{aligned}$$

with

$$\begin{aligned} d' (t) = \left\{ \begin{array}{ll} \Delta d \;\; &{}\text{ for } 0\le t\le \tau \\ 0 \;\; &{}\text{ otherwise } \end{array} \right. \end{aligned}$$

    Here $\mathscr {N}(0,1)$ denotes normal distribution with mean 0 and standard deviation 1. We do not make a choice of very high noise strength because it can make *r* negative which is physically meaningless. On the other hand, the noise should be substantially large enough to give rise to heterogeneity. A different value of noise other than 0.05 can also be chosen keeping in mind the above points and it will lead to similar results. It is also important to note that adding noise to different parameters also leads to similar results but we need to be careful about the non-negativity of parameters and as the value of *r* is greater than *p*, we apply the noise in the reproduction parameter. Also, as the death pulse is given in parameter *d*, we kept it outside the scope of adding noise here. We have found that for multiplicative lognormal noise, the results remain the same. This has been discussed in Sec. 4 of the Supplementary Material and shown in Figure S4 therein. The simulation of the system was run 1000 times with a step size of 0.001. One may note that we have not used the ordinary differential equations (ODE). This is because the coexistence cannot be observed in ODE as after long term evolution, it eventually converges to an absorbing point^33,68,70^ (See Sec. 3 in the Supplementary Material).

It is quite intriguing that anytime we apply a death pulse, we always observe one species living for a very long time. This has been shown in Supplementary Material Figure S3. In spite of the initial condition remaining the same, we start to see various species survive in different runs when we add a modest amount of white noise to the reproduction rate. Therefore it shows an apparent disagreement with the results obtained from Monte-Carlo simulations. To establish the credibility of this approach, we simulate the system without any death pulse and for identical initial conditions and find that for 1000 identical runs, species A survives in $33.1\%$ cases, species B in $34.3\%$ cases, and species C in $32.6\%$ cases. This matches with the fact that under equivalent conditions all three species have equal survival probability (small deviation is due to randomness). Instead of noise following normal distribution, uniform noise with the width 0.2 around reproduction rate has been simulated and found that species A survives in $32.7\%$ cases, species B in $34.9\%$ cases, and species C in $32.4\%$ cases signifying that symmetrically distributed noise will give rise to similar results. In the case of this stochastic approach, we find that results do not depend upon initial conditions. While simulating without any noise from identical parameters and identical initial conditions, we find all species’ densities to attain a steady state at the value of 0.22, which is the same as Monte Carlo simulations. The results obtained from the two approaches thus happen to be consistent with each other.

**Figure 4:**
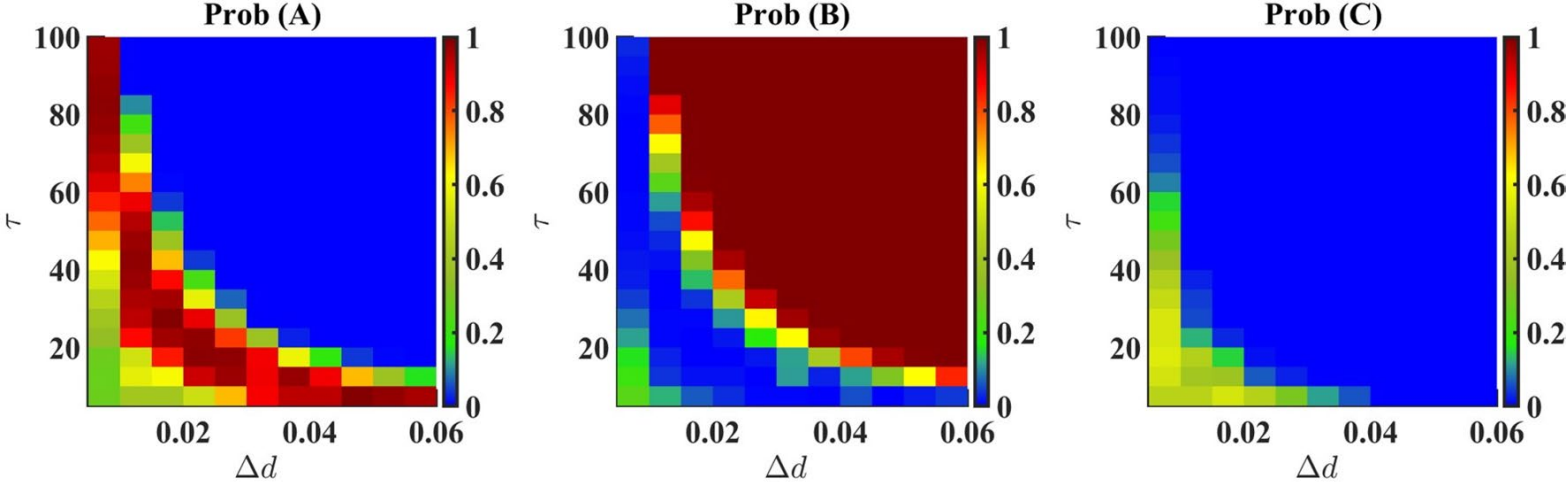
Probabilities of occurring (*A*, 0, 0) or (0, *B*, 0) or (0, 0, *C*) as a final state on $\Delta d - \tau$ plane obtained through numerical solution of stochastic differential equations. The results are almost consistent with the Monte-Carlo simulation (Fig. 3).

Under varying death rates (in species *A*) and time duration of the pulse, the results are summarized in Fig. 4. Here, the survival probability of a species denotes how many times it survives as a single species out of all the runs conducted. This result is in line with the result obtained through Monte Carlo simulation (Fig. 3) except for a scaling factor of 10 in the death pulse, $\Delta d$. Here, we have used a 10-times lower death rate than that of the Monte-Carlo simulation. Similar to Fig. 3, we have seen in Fig. 4 that species *B* is completely dominant for higher values of $\Delta d$ and $\tau$. Species *A* have greater survival probability at a small patch in the $\Delta d - \tau$ plane. For very small values of $\Delta d$ and $\tau$, we observe species C to be dominant, whereas, in the case of Monte-Carlo simulation, this region shows coexistence. Though we can not get coexistence from this method, still we see that in this region all the species have comparable probabilities of survival. This indicates the coexistence in an indirect way.

Effectively, the Monte-Carlo simulation that has been run for this system gives us a result that is averaged over a large equilibration time and over different initial configurations as well. Thus the results produced from the simulation may be thought of as an ensemble average. On the other hand differential equations only give us one realization of the ensemble. That is why we need to simulate the differential equations multiple times to obtain the ensemble average. This way the comparison of the results obtained from the two methods appears legitimate and consistent as well.

**Figure 5:**
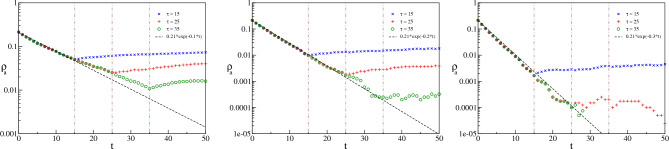
Dynamics inside the pulse: $\ln \rho _a$ vs time for $\tau = 15, 25, 35$ (marked by vertical dotted-dashed lines) having $\Delta d = 0.1, 0.2, 0.3$ for left, middle and right panel respectively and starting from the same initial configuration. The black dashed line shows the curve for exponential decay of the form $\rho _a^0 \exp (-\Delta d t)$, where $\rho _a^0 = 0.21$.

If we compare the results of probabilities of survival obtained from Monte-Carlo (MC) simulation in Fig. 4 and stochastic differential equations (SDE) in Fig. 5, we observe a difference of scaling of factor 10 in the values of $\Delta d$. In particular, the $\Delta d$’s in the case of SDE is 1/10th of that in the case of the Monte-Carlo simulation required for producing the same probabilities at the same $\tau$. To the best of our knowledge, we can affirm that this difference does not counter the consistency of the two results. It may originate due to the difference in the concept of time units in two completely different schemes. We have checked the results for many different parameters and observed that the difference of scaling always remains the same (see Figure S5 of Supplementary Material for more results).

## Deterministic decay of species *A* within pulse

Now let us focus on the dynamics of *A* within the pulse duration. Fig. 5 shows the variation of $\ln \rho _a$ against time for different pulse durations and heights of the death-pulse ($\Delta d$). The vertical lines in all three figures mark the three pulse durations, $\tau = 15, 25, 35$ on the time axis. The density of *A* shows an exponential decay for a small time after the commencement of the pulse. This can be understood analytically following the deterministic part of the Eq. 5 (i.e., excluding the random noise in the reproduction rate) for $t<\tau$. In this region, the dynamics of $\rho _a$ is mainly governed (at least initially) by $\Delta d$ because the boundary conditions

$$\rho _a (0)=\rho _a^0,\; \rho _b (0)=\rho _b^0,\; \rho _c (0)=\rho _c^0$$

i.e., the coexistent fixed-points make the term $(r \rho _v(t)- p\rho _c(t)- d) = 0$ (see Ref.^68^ for details). Therefore, for a small time, say $\Delta t$ after the commencement of the pulse, the dynamics of $\rho _a$ is described by the equation

6 $$\begin{aligned} \dfrac{\partial \rho _a}{\partial t} \approx - \Delta d \cdot \rho _a \end{aligned}$$

which brings about the decay of $\rho _a$ in an exponential Malthusian manner^27^ of the form

7 $$\begin{aligned} \rho _a (\Delta t) \approx \rho _a^0 e^{-\Delta d\cdot \Delta t} \end{aligned}$$

In the three cases of Fig. 5 we have plotted Eq. (7) alongside the numerical data with respective values of $\Delta d$. It is however to be noted that after a short time, the other species’ densities come into play and the numerical plots begin to deviate from the Malthusian manner.

## Conclusions

Our aim was to study the transient as well as the long-time behavior of a species as a part of cyclic interaction within an ecosystem when it was exposed to a sudden increase in the death rate and to check the possibility of survival with coexistence of the species. Our principal interest was to investigate the fate of a species and the entire ecosystem it belongs to if suddenly attacked by some disease or a natural calamity that might threaten its existence. By applying a pulse of death rate for a finite duration time to one of the species in a three-species rock-paper-scissor model we investigated the dynamics by Monte-Carlo simulation and later by numerical solution of stochastic differential equations. One of our main observations is an exponential decay of the affected species during the short duration of the pulse that could be determined analytically. Another important observation is in the long-time behavior when the affected species remains in the system as a lone survivor for certain strengths and durations of the death pulse.

While the exponential decay and its deviation shortly after the withdrawal of the pulse can be understood analytically from the differential equation, the atypicality lies in the long-time time dynamics when it upholds species *A* as the only survivor. This is attributed to the abolition of species *C* before the other two. We see that for a range of the values of $\tau$ and $\Delta d$, coexistence remains intact in the long time limit. This means that the disturbance in these cases are not strong enough and the parameters *d*, *r* and *p* are able to recover the coexistence. However, if $\tau$ (or $\Delta d$) is slightly increased, both *A* and *C* become susceptible to the abolition: The decay of *A* is ascribed to the increase of its death rate, whereas the decay of *C* is due to the growth of predation of *B* (because *B* gets privilege as *A* dies out). In this situation, there is a probability that *C* is abolished prior to *A*. When it happens, *A* having no predator, diminishes *B* and becomes the only survivor in the long run. This probability decreases with increasing $\tau$ (or $\Delta d$) and for large $\tau$ and $\Delta d$, species *A* always dies out first keeping *B* and *C*. Then *B* survives eventually wiping out *C*.

Question may arise on the role of the parameters *d*, *r*, and *p*. These parameters are significant in bringing about as well as maintaining coexistence. We have to start with a set of $\{ d,r,p \}$ for which the coexistence can be achieved. Otherwise, the system would go to a single-species state and the application of death-pulse would have no meaning then. Even after the pulse is over, these parameters try to bring back coexistence within the system. Therefore the effects of the pulse parameters ($\tau$ and $\Delta d$) actually counter the effects of $\{ d,r,p \}$ and try to jeopardize coexistence. For any set of $\{ d,r,p \}$ that results in coexistence, the effect of the pulse is the same.

This simple model may be useful in studying the aftermath of an ecosystem facing a disease spread, natural calamities, or any other catastrophe that usually persists for a finite duration of time. An estimate of the strength and the duration of the perturbation may be obtained upto which the ecosystem can revive its biodiversity. In this connection, this model may help study and innovate revival strategies for an ecosystem under such exemplary situations.

### Supplementary Information

Supplementary Information.

## Data Availability

The datasets used and/or analysed during the current study available from the corresponding author on reasonable request.
