# Supplementary material for: Response of a three-species cyclic ecosystem to a short-lived elevation of death rate
Source: Sci Rep. 2023 Nov 25;13:20740. doi: 10.1038/s41598-023-48104-6 (PMC10676407; doi:10.1038/s41598-023-48104-6)
Supplement: Supplementary file 1 — Supplementary Information. [file 41598_2023_48104_MOESM1_ESM.pdf]

Supplementary Material for Response of a three-species cyclic ecosystem to a short-lived elevation of death rate

Sourin Chatterjee[1,+], Rina De[2,+], Chittaranjan Hens[3,4], Syamal K. Dana[4,5], Tomasz Kapitaniak[4], and Sirshendu Bhattacharyya[2,*]

[1]Department of Mathematics and Statistics, Indian Institute of Science Education and Research, Kolkata, West Bengal 741246, India
[2]Department of Physics, Raja Rammohun Roy Mahavidyalaya, Radhanagar, Hooghly 712406, India
[3]Center for Computational Natural Sciences and Bioinformatics, International Institute of Information Technology, Gachibowli, Hyderabad 500 032, India
[4]Division of Dynamics, Faculty of Mechanical Engineering, Lodz University of Technology, 90-924 Lodz, Poland
[5]Centre for Mathematical Biology and Ecology, Department of Mathematics, Jadavpur University, Kolkata 700032, India
[*]sirs.bh@gmail.com
[+]these authors contributed equally to this work

1 Time series for a different reproduction rate

The temporal behavior of the densities have been observed and presented in Fig. S1 for a different value of the reproduction rate ($r = 0.6$). The value has been so chosen that the criteria of coxistence remain intact. As a result, the initial densities become different from the case shown in Fig. 2 of main text but the observation remains the same.

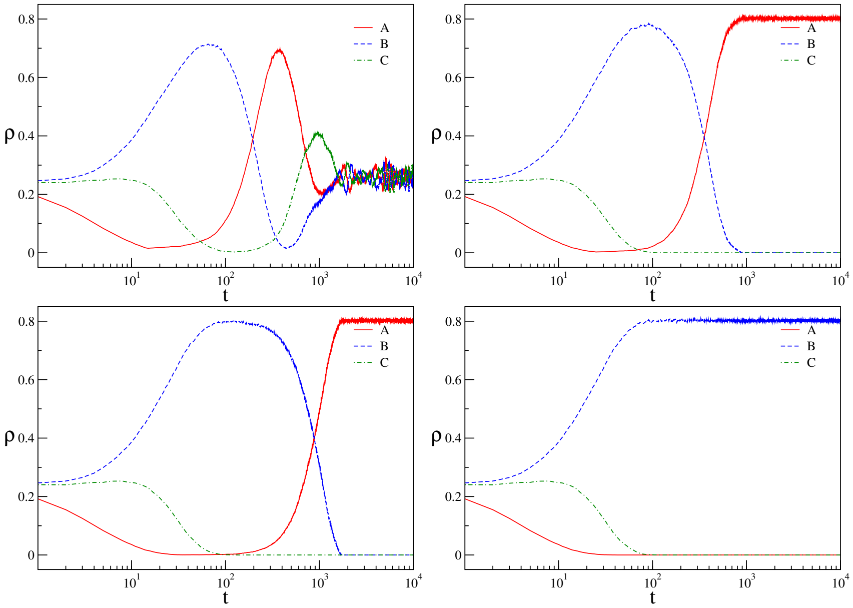

Figure S1. Real time dynamics of the densities of A (red line), B (blue dashed line), and C (green dot-dashed line) for different τ (15, 25, 35, 45 from upper left to lower right). In all the cases, $d = 0.1$, $p = 0.2$, $r = 0.6$ and $\Delta d = 0.2$.

2 Results for larger time and larger system sizes

In Fig. S2 we have demonstrated the results that have been checked for larger time as well as larger system sizes. The time units of observation have been extended upto 10^5. In addition to $N = 200$, results have been checked for $N = 300$ and 400. The main observation remains the same in these cases.

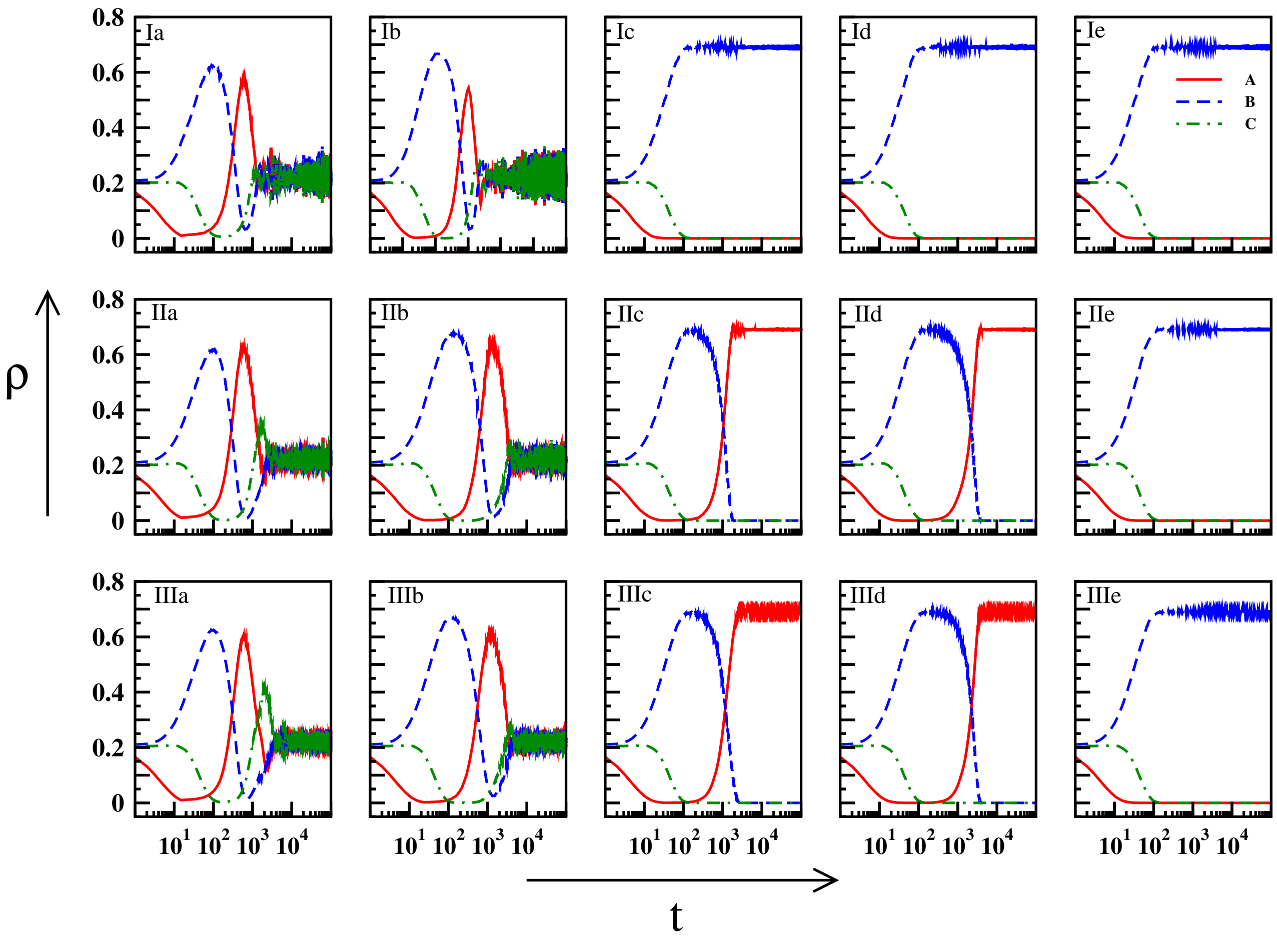

Figure S2. Density vs time plots for different system sizes. Densities of A, B and C are shown in red line, blue dashed line and green dot-dashed line respectively. Three rows (I, II, III) are for three different system sizes: $N = 200, 300, 400$ respectively, and five columns (a - e) are for five different values of τ: 15, 25, 35, 45, 55. In all the cases, $d = 0.1$, $p = 0.2$, $r = 0.4$ and $\Delta d = 0.2$. Dynamics of all the densities are shown upto 10^5 time units.

3 Results from ordinary differential equations

The model of death pulse has been simulated using ordinary differential equations (ODE) for identical initial conditions i.e., starting from identical densities of all the species shown in Fig. S3. The results show that the coexistence can never be achieved using this scheme.

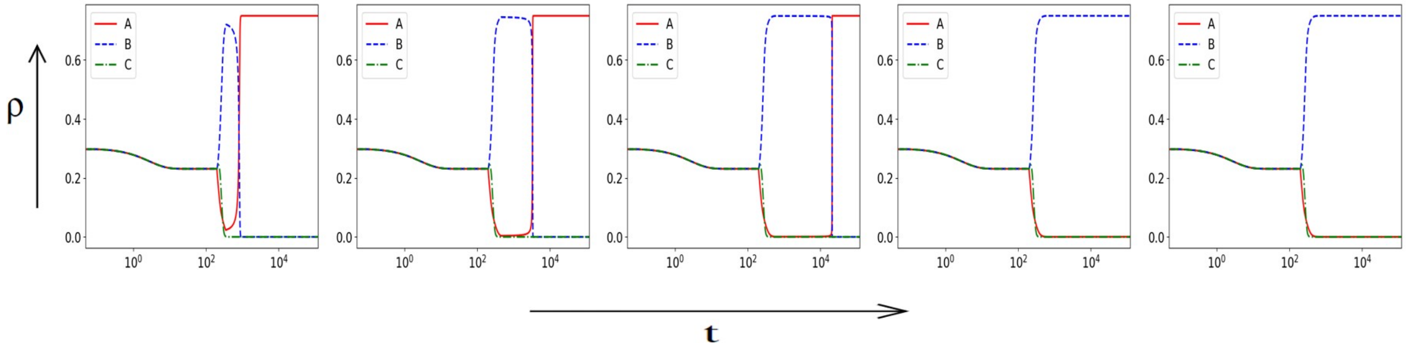

Figure S3. Density vs time plots for $\tau = 15, 25, 35, 45, 55$ (from left to right). In all the cases, $d = 0.1$, $p = 0.2$, $r = 0.4$ and $\Delta d = 0.02$. The results are plotted using deterministic version of the stochastic differential equations for our model. The extreme left plot suggests that one species (species A) is stable after long term evolution. The coexistence appearing in MC simulations is absent here. However, for larger τ, the result of MC, SDE and ODE remain similar: Only species B survives.

4 Results for multiplicative lognormal noise

We have checked the results for multiplicative lognormal noise instead of additive normal noise and found it does not affect the results. So, the Eq. 5 in the main text changes to:

$$
\begin{aligned}
\frac{d\rho_a}{dt} &= \rho_a(t)\left[(r\mathscr{X})\rho_v(t) - p\rho_c(t) - (d+d')\right] \\
\frac{d\rho_b}{dt} &= \rho_b(t)\left[(r\mathscr{X})\rho_v(t) - p\rho_a(t) - d\right] \\
\frac{d\rho_c}{dt} &= \rho_c(t)\left[(r\mathscr{X})\rho_v(t) - p\rho_b(t) - d\right] \qquad \text{(S1)}
\end{aligned}
$$

with

$$
d'(t) = \begin{cases} \Delta d & \text{for } 0 \le t \le \tau \\ 0 & \text{otherwise} \end{cases}
$$

$$
\text{and} \quad \ln(\mathscr{X}) \sim \mathscr{N}(0,1)
$$

We find that from identical initial conditions and without any death pulse species A survives in 34.6% of cases, species B in 33.7% of cases, and species C in 32.7% of cases quite similar to the result for additive normal noise. In Fig. S4 we have shown three scenarios where three different species dominate.

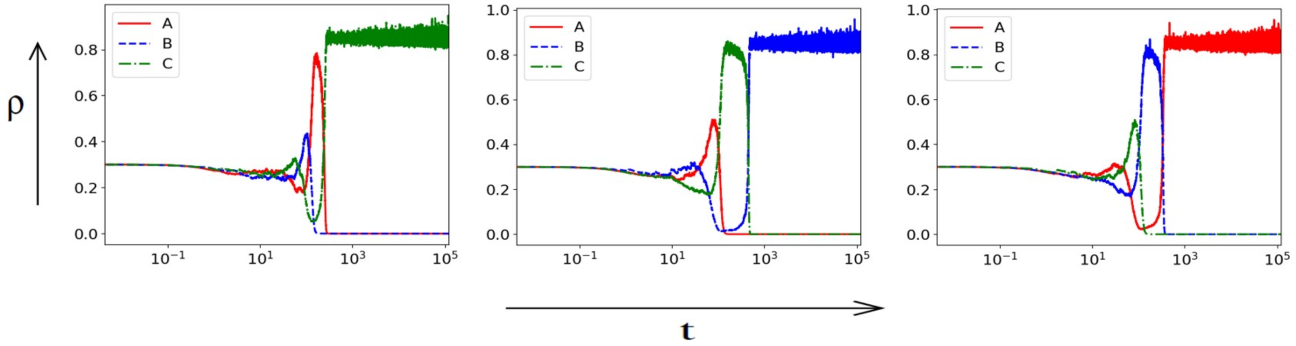

Figure S4. Three exemplary scenarios in applying multiplicative lognormal noise.

5 Comparison of results obtained from two schemes

We have shown below (in Fig. S5) two comparative pictures of the probabilities of survival of single species B as obtained from MC and SDE for two different values of reproduction rate (r), keeping τ fixed (at 40). The difference of scale in Δd obtained from two different schemes always remain the same. This consistency assures the agreement of the results from two schemes.

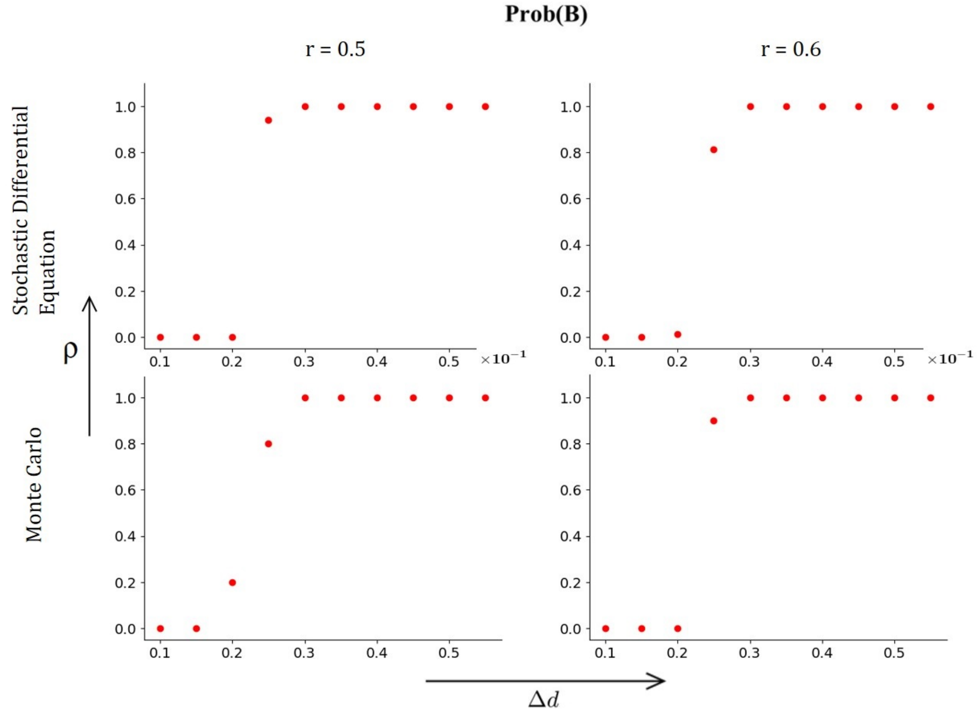

Figure S5. Pr(B) vs Δd time plots for $\tau = 40$. The two columns are for two different reproduction rates: $r = 0.5$ and 0.6. The upper row presents the results obtained from SDE and the lower row presents that from MC. The values of Δd (on x-axes) on the upper row is $1/10$th of that on the lower row.
